# Kinesthetic imagery training of forceful muscle contractions increases brain signal and muscle strength

**DOI:** 10.3389/fnhum.2013.00561

**Published:** 2013-09-26

**Authors:** Wan X. Yao, Vinoth K. Ranganathan, Didier Allexandre, Vlodek Siemionow, Guang H. Yue

**Affiliations:** ^1^Department of Health and Kinesiology, University of Texas at San AntonioSan Antonio, TX, USA; ^2^Department of Physical Medicine & Rehabilitation, Neurological Institute, The Cleveland ClinicCleveland, OH, USA; ^3^Department of Biomedical Engineering, Lerner Research Institute, The Cleveland ClinicCleveland, OH, USA; ^4^Human Performance and Engineering Laboratory, Kessler Foundation Research CenterWest Orange, NJ, USA

**Keywords:** motor imagery training, muscle strength, electroencephalography (EEG), movement-related cortical potential (MRCP)

## Abstract

The purpose of this study was to compare the effect of training using internal imagery (IMI; also known as kinesthetic imagery or first person imagery) with that of external imagery (EMI; also known as third-person visual imagery) of strong muscle contractions on voluntary muscle strengthening. Eighteen young, healthy subjects were randomly assigned to one of three groups (6 in each group): internal motor imagery (IMI), external motor imagery (EMI), or a no-practice control (CTRL) group. Training lasted for 6 weeks (~15 min/day, 5 days/week). The participants' right arm elbow-flexion strength, muscle electrical activity, and movement-related cortical potential (MRCP) were evaluated before and after training. Only the IMI group showed significant strength gained (10.8%) while the EMI (4.8%) and CTRL (−3.3%) groups did not. Only the IMI group showed a significant elevation in MRCP on scalp locations over both the primary motor (M1) and supplementary motor cortices (EMI group over M1 only) and this increase was significantly greater than that of EMI and CTRL groups. These results suggest that training by IMI of forceful muscle contractions was effective in improving voluntary muscle strength without physical exercise. We suggest that the IMI training likely strengthened brain-to-muscle (BTM) command that may have improved motor unit recruitment and activation, and led to greater muscle output. Training by IMI of forceful muscle contractions may change the activity level of cortical motor control network, which may translate into greater descending command to the target muscle and increase its strength.

## Introduction

Accumulating evidence suggests that mental training without physical or muscle exercise can improve voluntary muscle strength (Yue and Cole, 1992; Yue et al., 1996; Smith et al., 2003; Zijdewind et al., 2003; Ranganathan et al., 2004; Sidaway and Trzaska, 2005; Shackell and Standing, 2007). This finding could have significant application in rehabilitation medicine (Jackson et al., 2001) because numerous weak patients or frail older adults who find it difficult or unsafe to participate in conventional strength training (such as weightlifting) programs, may now be able to strengthen their muscles by using their mind. It has been shown that the main underlying mechanism for motor imagery (MI) training-induced strength gains is by adaptations occurring in the nervous system. For example, after 4 weeks of mental training, the strength of the little finger abduction force increased 22%; the augmentation accompanied an increase in the electromyographic (EMG) signal of the finger abductor that represented overall neural input to the muscle (Yue and Cole, 1992). In another study, two groups of volunteers had their little finger of the left hand immobilized for 4 weeks during which one group performed MI training of maximal voluntary contractions (MVC) and the other [control (CTRL) group] did not. After immobilization, both groups showed muscle atrophy but strength reduction only occurred in the CTRL group. The MI group maintained the strength with a significant increase in the EMG signal despite muscle atrophy caused by immobilization (Yue et al., 1996). In this case, the increase of neural (EMG) signal appeared to compensate for strength loss due to the atrophy. More recently, Ranganathan et al. (2004) demonstrated MI training-induced strength gains in a finger and upper-arm muscle that accompanied an elevation in the cortical signal directly related to the execution of strength-production muscle contractions. These observations support the hypothesis that the descending command from the brain to target muscle for MVC can be strengthened by MI training alone, which in turn increases maximal muscle force by recruiting additional motor units and/or increasing activation level of the participating motor units.

Despite finding of significant strength gains by MI training in a majority of studies in this area, one investigation (Herbert et al., 1998) did not reported similar results. In this study, no significant strength gain specifically associated with MI training was observed following an 8-week training program. The discrepancy in the results between this (Herbert et al., 1998) and other MI strength training studies could have been caused by different imagery procedures adopted by the investigators. There are two common types of mental imagery—internal and external imagery. In internal imagery (IMI; also known as kinesthetic or first-person imagery), a person imagines or mentally creates the feeling of performing the exercise from within the body (i.e., from a first-person perspective). For example, mental strength training using internal imagery emphasizes that the subject generates a similar feeling as he/she felt during a physical MVC (e.g., Ranganathan et al., 2004; Sidaway and Trzaska, 2005). In external imagery (EMI; also known as third-person visual imagery), the person sees or visualizes performing the task from outside the body—similar to watching oneself in a mirror performing an exercise (i.e., from a third-person perspective). Performing IMI generates significantly more physiological responses [such as heart rate (HR), blood pressure (BP), and respiration rate] compared to doing EMI (Ranganathan et al., 2004). Many studies (Mumford and Hall, 1985; Murphy, 1994; White and Hardy, 1995; Reed, 2002) have indicated that IMI is superior to EMI for improving motor skills. Studies have reported that highly skilled athletes predominantly use IMI to enhance their performance (e.g., Roure et al., 1998). It is possible that participants in the study of Herbert et al. (1998) adopted EMI procedure for the mental training, which did not result in a significant strength gain. For those studies that demonstrated significant strength increases, the MI training procedure was clearly using IMI (Yue and Cole, 1992; Yue et al., 1996; Smith et al., 2003; Zijdewind et al., 2003; Ranganathan et al., 2004; Sidaway and Trzaska, 2005; Shackell and Standing, 2007). However, no studies have attempted to compare the effects of two (internal and external) imagery training regimens on muscle strengthening. The purpose of this study was to compare strength gains following the two mental training programs: IMI and EMI.

## Methods

### Subjects

Eighteen right-handed young (18–35 years) and healthy volunteers were recruited and randomly assigned to three groups: internal motor imagery (IMI), external motor imagery (EMI), and no-practice CTRL groups. None of the subjects had participated in any regular exercise program for at least a year prior to the study. The training lasted for 6 weeks (15 min/day for 5 days/week). The Institutional Review Board at the Cleveland Clinic approved the study and all participants gave their informed consent prior to participation.

### Training protocol

Subjects in the IMI group imagined performing the task from a first-person perspective, i.e., they visualized and feel as if they were physically executing a maximal elbow-flexion contraction (Ranganathan et al., 2004). During each trial, they were instructed to imagine their forearm pushing maximally upward against the force transducer that was used for the strength measurements during the pre-training tests or against a heavy object. In other words, they *urged* the elbow-flexor muscles to contract maximally during each training trial. Some participants visualized putting the forearm under a heavy table and then tried very hard mentally to lift the table. Subjects could see the stationary arm held on the side of the body when imagining the contraction even though many subjects performed the mental exercises with their eyes closed. This same IMI protocol was found to significantly elevate HR and BP in a prior study (Ranganathan et al., 2004). Subjects in the EMI group viewed (in their mind) themselves performing the elbow flexion task from a third-person perspective, i.e., they watched themselves performing the task in their mind without a strong intent to make the contraction. In each 15-min training session, subjects performed 30 training trials, ~15-s per trial followed by a 15-s rest. EMG signals from the two elbow flexor muscles, biceps brachii, and brachioradialis that are accessible from skin surface were viewed in every trial and session and recorded randomly in some trials and sessions to monitor activity level of the target muscles. On average, the EMG amplitude during the IMI and EMI training was less than 2% EMG for the MVC performed during the pre-training strength test (see below) and did not differ between the two training groups.

### Force (strength) measurement

Right arm elbow flexion force was measured by a force transducer (JR3 Universal Force-Moment Sensor System, Woodland, CA) with subjects seated, their right hand placed in a wrist cuff, forearm in a neutral position and an elbow joint angle of ~ 100° (Ranganathan et al., 2004). The elbow was supported at hip height and the shoulders and torso were kept in position using restraints. Three elbow flexion MVC trials were performed in each measurement session and the highest force among the trials was analyzed. For each trial, participants were verbally encouraged to exert maximal force. Strength measurements were made before and after the 6-week training period. The strength measurement conditions (arm and body positions), and joint angles were carefully measured each time and maintained over the sessions. In addition, the verbal instruction and encouragement for maximal force production were the same for all measurement sessions. The force data was digitized at 100 samples/s using a data acquisition system (Micro 1401, Cambridge Electronic Design, Ltd., Cambridge, UK) and recorded on hard drive of a personal computer (PC).

### EMG measurement

Surface EMG was recorded during the elbow-flexion MVC force measurement trials using bipolar surface electrodes (Ag-AgCl, In Vivo Metric, Healdsburg, CA; 8-mm recording diameter and 2 cm apart of the two electrodes) from the belly of the biceps brachii (BB) and triceps brachii (TB) muscles. A reference electrode was placed on the skin overlying the lateral epicondyle near the elbow joint. Average BB EMG during a period when the MVC force was stable in each trial was calculated and the trial that yielded the highest average EMG was analyzed. The TB EMG during the elbow flexion MVCs was normalized to the TB EMG recorded during the elbow extension MVC and was a measure of the antagonist (TB) muscle activity during strength performance of the agonist (elbow flexor) muscle group. The EMG signal was amplified (× 1000) and band-pass filtered (3 Hz–1 KHz) using a Neurodata Amplifier system (Model 15A54, Grass Instrument Co., Quincy, MA), digitized (2000 samples/s) using the Micro-1401 system, and recorded on hard drive of the PC.

### EEG and MRCP measurement

EEG electrodes were placed on the scalp roughly overlying the supplementary motor area (Cz), contralateral (C3) and ipsilateral (C4) sensorimotor regions, and central location of the frontal lobe (Fz). Electrode locations were determined based on the International 10–20 System (Jasper, 1958). Conducting gel (Electro-gel™, Electro-Cap International, Inc., Eaton, OH) was injected into each electrode to connect the recording surface of the electrode with the scalp. Impedance between each electrode and the skin was maintained below 5000 Ω (at 30 Hz). The EEG signal was amplified (× 20,000) and band-pass filtered (0.1–100 Hz) by the Neurodata Amplifier system, digitized (500 samples/s) using the Micro-1401 system, and stored on hard disk of the PC.

In each measurement session, the EEG recording was made after the strength and associated EMG data collection. Participants performed 30-elbow flexion MVCs (once every 20 s) during the EEG recording. The purpose of performing multiple MVC trials was to obtain triggered averaging of the MVC-related cortical potential (MRCP) with improved signal-to-noise ratio. Raw EEG data were visually examined, and trials with artifacts (such as eye blinks) were excluded. For each EEG-MVC trial, a 4-s window of the EEG was triggered by the force output (threshold = 5% initial MVC force), 2 s before and 2 s after the trigger (Siemionow et al., 2000; Fang et al., 2001). The triggered averaging (over the 30 trials) was performed by Spike 2 data analysis software (Cambridge Electronic Design, Ltd., Cambridge, UK) associated with the Micro-1401 system. The amplitude of each averaged MRCP was measured from the baseline to the peak of the negative potential (to view the shape of MRCP and its measurement, see Figures in: Siemionow et al., 2000; Yue et al., 2000; Fang et al., 2001). Because the MRCP was time-locked to each MVC, it was considered directly related to the planning and execution of the MVC. It has also been shown that there is a direct linear relation between force strength, EMG signals during voluntary muscle activation, and the amplitude of MRCP (Siemionow et al., 2000). Thus, increases in MRCP amplitude after training can be considered a direct indication of an enhancement in the descending command to the target muscle (Ranganathan et al., 2004).

### Statistical analysis

One-Way analysis of variance (ANOVA) was used to test for group difference at baseline for all outcomes. For all analyzes, group (IMI, EMI, and CTRL) was chosen as the independent variable and percentage MVC, EMG, and MRCP changes from baseline as the dependent variables. The choice of using percentage change was made to adjust for inter-individual baseline difference. Within group comparison was first performed to test for significant percentage change using one group student *t*-test comparison. Then ANOVA was used to test for overall between group comparisons (equivalent to a group by time interaction) followed by *post-hoc* analyzes to perform pair-wise group comparison. Given the pilot nature of the study, to avoid Type II error significance levels are first presented without correcting for multiple comparisons. Implication of performing significance level adjustment using the conservative Sidak method is then presented and its implication discussed. Given the small sample size and thus, potential strong influence of small deviation from normality, sensitivity analysis was also performed by running non-parametric Kruskal-Wallis and Wilcoxon tests for overall and pair-wise group comparisons.

The level of significance was set at 0.05 for all statistical analyzes. Results are given as mean ± SE. All analyzes were conducted using IBM SPSS version 21 and Excel for simple *t*-test.

## Results

### Baseline comparison

No significant between group differences were found for strength [*F*_(2, 15)_ = 1.6, *p* = 0.23], and BB [*F*_(2, 15)_ = 1.22, *p* = 0.33] and TB [*F*_(2, 15)_ = 0.86, *p* = 0.44] EMG.

### Changes in strength after training

**Figure 1:**
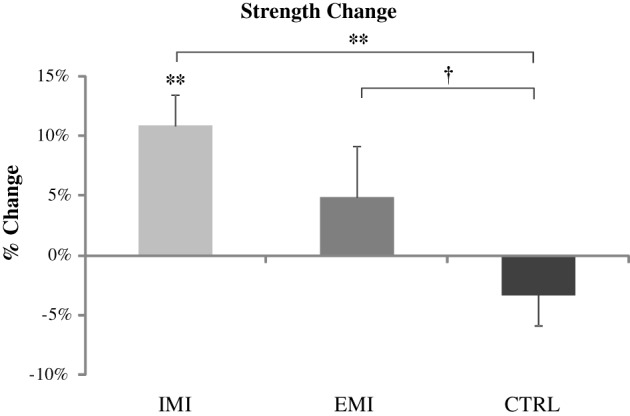
**Pre- to post-training percentage change in strength values for all three groups**. Only the IMI group had a significant strength gain after training which was significantly greater than Control. EMI shows only marginal greater strength gain compared to Control. ^†^*P* < 0.1, ^*^P < 0.05, ^**^P < 0.01.

The IMI group had significant strength gains [mean ± *SE*, 10.8 ± 2.7%, *t*_(1, 5)_ = 4.06, *P* = 0.01] after the 6-week training while the change in strength in the EMI group after training was not significant [4.8 ± 4.3%, *t*_(1, 5)_ = 1.13, *P* = 0.31; Figure 1. Subjects in the CTRL group who did not perform training of either kind, had no strength gain [−3.3 ± 2.61%, *t*_(1, 5)_ = 1.25, *P* = 0.27]. Using ANOVA, a significant group effect was found on the percentage change in strength, *F*_(2, 15)_ = 4.66, *P* = 0.03. Compared to control, *post-hoc* tests reveal the improvement for IMI to be significant compared to control, *t*_(1, 15)_ = 3.04, *P* = 0.008 while for EMI the improvement was only marginal compared to control, *t*_(1, 15)_ = 1.75, *P* = 0.10. Group difference between EMI and IMI was not significant, *t*_(1, 15)_ = 1.29, *P* = 0.22.

Furthermore, all participants in the IMI group (compared to only 50% in the EMI group) showed clinically meaningful strength gains (defined as being of medium effect size based on Cohen's d definition i.e., percentage change greater than half the overall standard deviation which is equal to 9.6%). The between group difference in the percentage of participants who improved were close to significance (*P* = 0.09 using Fisher's exact test). The strength results suggest that the IMI training can significantly improve maximal elbow flexor muscle force from baseline but the EMI training cannot.

### Changes in BB and TB EMG

**Figure 2:**
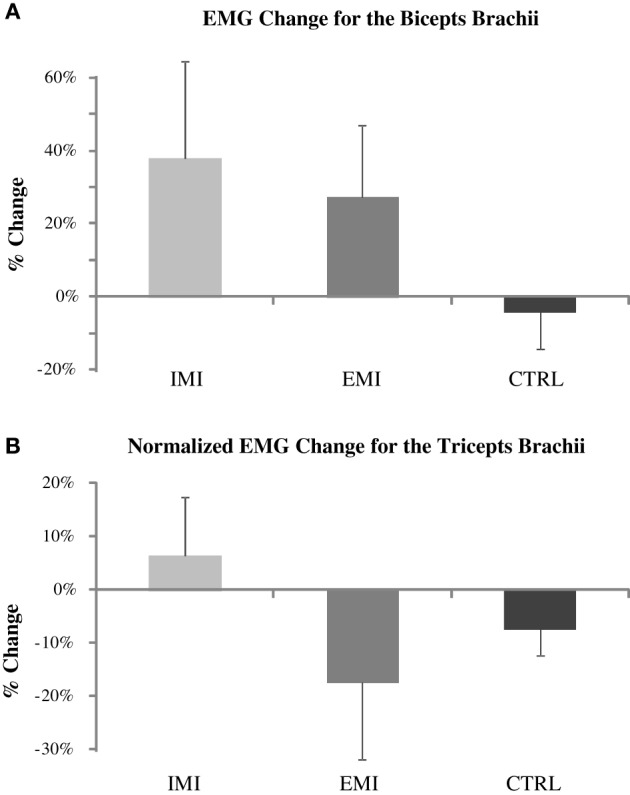
**Pre- to post-training percentage change in the biceps brachii (BB) average EMG (A) and normalized triceps brachii (TB) EMG (B) for IMI, EMI, and CTRL groups**. The TB EMG during elbow flexion MVC was normalized to TB EMG during elbow extension MVC. The apparent BB EMG increases for the IMI and EMI training (upper panel) were not significant due to large data variations.

Along with increases in strength, muscle electrical activity (EMG) of the BB muscle increased by 38 ± 26% for the IMI group and by 27 ± 19% for the EMI group but due to the huge variation in EMG values across subjects (standard deviation of 65 and 47%, respectively), these increases were not significant [*t*_(5)_ = 1.43, *p* = 0.21 and *t*_(5)_ = 1.42, *p* = 0.22, respectively] (Figure 2A). The normalized TB EMG did not change significantly either with an increase of 6 ± 11% for IMI (*p* = 0.58) and decrease of 17 ± 15% for EMI (*p* = 0.28) (Figure 2B). The CTRL group had minimal changes in EMG before and after the training period (Figure 2). Similarly ANOVA showed no overall group effect for both percentage change in BB EMG [*F*_(2, 15)_ = 1.22, *P* = 0.32] and TB EMG [*F*_(2, 15)_ = 1.21, *P* = 0.33].

### Changes in MVC-related cortical potential (MRCP)

**Figure 3:**
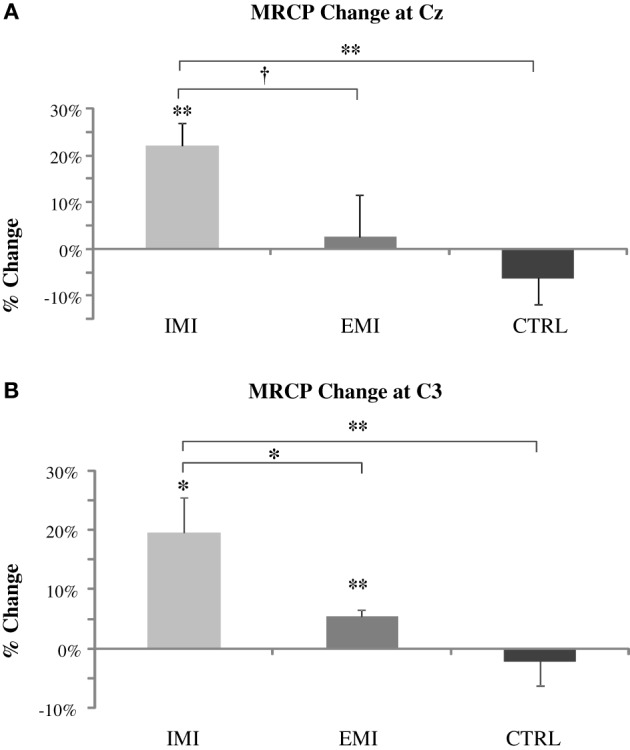
**Pre- to post-training percentage change in MRCP amplitude at Cz (roughly over the supplementary motor area) (A) and C3 (roughly over the contralateral sensory motor area) (B) locations**. The IMI group had a significant increase in the MRCP amplitude at Cz and C3 and EMI at C3 only while no significant change was observed for CTRL groups did not. MRCP increases for IMI were significantly greater than control for Cz and C3 and greater than EMI for C3 and marginally so for Cz. EMI increases compared to Control were not significant. ^†^*P* < 0.1, ^*^P < 0.05, ^**^P < 0.01.

Subjects in the IMI, EMI, and CTRL groups performed 30 MVC trials before and after training, and MRCP was derived by triggered-averaging of the EEG data associated with the MVCs. Figure 3 shows MRCP percentage change for both the Cz and C3 locations. The baseline-to-peak MRCP value at Cz and C3 electrode locations significantly increased by 22 ± 5% [*t*_(5)_ = 4.48, *P* = 0.007] and 20 ± 6% [*t*_(5)_ = 3.34, *P* = 0.02], respectively for the IMI group, and 2.6 ± 9% [*t*_(5)_ = 0.29, *P* = 0.79] and 5.4 ± 1.2% [*t*_(5)_ = 4.41, *P* = 0.007] for EMI while the no-practice CTRL groups did not have any significant changes [−6.2 ± 5.6%, *t*_(5)_ = 1.11, *P* = 0.32 and −2.1 ± 4.0%, *t*_(5)_ = 0.53, *P* = 0.62]. Note that even though C3 MRCP changes for EMI were statistical significant, this was mainly the result of an unusual small variation in the data (std = 3.0). The amplitude increase (2.6%) was comparatively smaller than EMI (22%) and thus, could be considered marginal. A significant group effect for both the Cz and C3 locations was found by running ANOVA on the percentage MRCP changes [*F*_(2, 15)_ = 4.59, *P* = 0.03 and *F* = 7.01, *P* = 0.01, respectively]. *Post-hoc* analyzes revealed that MRCP increases for IMI were significantly greater than CTRL for Cz [*t*_(1, 15)_ = 2.96, *P* = 0.01] and C3 [*t*_(1, 15)_ = 3.69, *P* = 0.002] and significantly greater than EMI for C3 [*t*_(1, 15)_ = 2.41, *P* = 0.03] and marginally so for Cz [*t*_(1, 15)_ = 2.04, *P* = 0.06]. No difference existed between EMI and CTRL [*t*_(1, 15)_ = 2, *P* = 0.37 for Cz and *t*_(1, 15)_ = 2, *P* = 0.53 for C3].

The slope or rate of force production during the MVC performance (for MRCP measurement) was similar before vs. after training and between groups; this ensured that the difference seen in MRCP amplitude between the groups was not caused by a discrepancy in the rate of force development (Siemionow et al., 2000).

### Correction for multiple comparisons and sensitivity analysis using non-parametric tests

When performing significance level adjustment to *post-hoc* analyzes using the conservative Sidak method, results remained significant for all outcomes except for IMI within group change for Cz MRCP which became borderline significant (*P* = 0.06) and between group changes for IMI vs. EMI C3 MRCP which became non-significant (*P* = 0.11).

The non-parametric Kruskal-Wallis and Wilcoxon tests for overall and pair-wise group comparisons showed a similar pattern of significance than ANOVA and *t*-test providing confidence that results are not the product of statistical or data distribution anomalies.

## Discussion

The primary finding of this study was that training involving internal mental imagery of strong muscle contractions significantly improved voluntary muscle strength but external mental imagery of the same motor task did not yield such strength increase. The strength augmentation in the IMI group was accompanied by a significant elevation in the level of brain activation (MRCP) compared to the baseline and MRCP change experienced by the EMI group. It is worth noting that EMG signals from the major elbow flexor muscle was monitored during every training session and the muscle activity remained well below 2% maximal contraction level and no difference was found in the EMG activity level during the training between the two groups.

Our findings indicate that the central nervous system reacts to IMI and EMI training differently. While both methods require the subject's attention (with negligible physical activity), MRCP results suggest that the IMI activates motor cortical areas [perhaps somatosensory areas (SMA) and M1] more than external imagery, probably because the cortical centers try to recreate the kinesthetic feeling and generate a strong command during the imagery. We suggest that this process might reinforce the neural circuitry and send stronger signals to the target muscle. This hypothesis of differential mode of action between IMI and EMI seems to be supported by recent imaging studies. Similarly to our findings, it has been found that IMI more greatly implicates motor related areas such as cerebellum, SMA, dorsal premotor cortex, and cingulated motor area than EMI does (Ruby and Decety, 2001; Naito et al., 2002; Malouin et al., 2004). Furthermore, IMI shows greater activation in the parietal and more specifically the inferoparietal cortex (Ruby and Decety, 2001; Naito et al., 2002; Malouin et al., 2004), areas known to be implicated in the sensory-visual representation and preparation of movement (Fogassi and Luppino, 2005), and thus, more likely to be involved in the IMI process than EMI where there was no intention to create movement. Our findings and that of others indicate that IMI more greatly activates motor regions involved in the planning and execution of movement than EMI, providing potential neural mechanisms underlying strength gains observed only in IMI.

The strength improvements accompanied an increase in time-locked cortical potential. This finding suggests that repetitive strong intention to maximally activate the elbow flexors trained and enabled the relevant brain regions to generate stronger signals to muscle. The relatively consistent MRCP values in the CTRL group (Figure 3; no significant percentage changes) before and after training suggests that the MRCP measurement is reliable even across many sessions and a long period of time as found in our prior study (Ranganathan et al., 2004). Previous research (Dettmers et al., 1995; Siemionow et al., 2000; Dai et al., 2001) has shown a proportional relationship between magnitude of brain-to-muscle signal and voluntary muscle force by young human subjects, indicating that greater strength is a consequence of stronger brain activity. A descending command could recruit the motor units that were otherwise inactive in an untrained state and/or drive the active motor units to higher intensity (higher discharge rate), leading to greater muscle force. Alternatively, the trained control network may be able to more effectively remove or reduce inhibitory input to the motoneuron pool of the muscles, resulting in a net increase in motoneuron output. Training-induced neural adaptations may also include improvements in muscle coordination, such as reductions in the activity of the antagonist muscles when performing the agonist muscle MVC (Carolan and Cafarelli, 1992). However, our EMG result from the TB muscle, antagonist of the elbow flexors did not change after training, indicating that the antagonist muscle did not play a significant role in the elbow flexion strength gain.

The sample size of this pilot study was small (6 in each group). Nevertheless, the consistent results obtained using parametric and non-parametric methods as well as contingency analysis on the percentage of participants who improved force strength provide sufficient confidence on the results obtained in this pilot study. Future studies with larger sample size would need to replicate this study to confirm the results and in particular demonstrate statistical significance between IMI and EMI. There was no objective method to monitor cortical activities during internal and EMI performances. Identifying an accurate and reliable brain signal that can be monitored online would not only enable the performer to more correctly carry out a given imagery task, but the signal may also be used for other purposes such as controlling an assistive device for rehabilitation via brain-computer or brain-machine interface. Given the non-local nature of EEG signals, the contribution of far-field effect to the results observed at C3 and Cz cannot be ruled out. High density EEG data will be needed to confirm that the observed activity changes come indeed from the supplementary and sensorimotor regions. Furthermore, the possibility of doing mapping and source localization of cortical potentials with high density EEG recording could also provide more information about the differences and mechanisms underlying various imagery training approaches. Adoption of functional imaging such as fMRI or PET during imagery exercise may provide additional information regarding location and activation level in the brain while performing internal imagery vs. external imagery tasks. Further research in this area is needed to overcome these limitations and better understand the differences in imagery perspectives and effect of various imagery training programs on strength and motor skill gains.

This study is the first to directly compare efficacies by IMI and EMI training regimes on muscle strength. Knowing that the IMI training is superior to EMI in gaining strength from baseline and generate greater descending command, the information is valuable to potentially provide guidance in implementing mental imagery training in clinical or sport environment. The findings have clinical importance to potentially adopt IMI training as a therapy to treat weakness in frail patients and older adults without undergoing intimidating conventional strength training.

### Conflict of interest statement

The authors declare that the research was conducted in the absence of any commercial or financial relationships that could be construed as a potential conflict of interest.
